# *Nosema* Tolerant Honeybees (*Apis mellifera*) Escape Parasitic Manipulation of Apoptosis

**DOI:** 10.1371/journal.pone.0140174

**Published:** 2015-10-07

**Authors:** Christoph Kurze, Yves Le Conte, Claudia Dussaubat, Silvio Erler, Per Kryger, Oleg Lewkowski, Thomas Müller, Miriam Widder, Robin F. A. Moritz

**Affiliations:** 1 Institute for Biology, Martin-Luther-Universität Halle-Wittenberg, Halle (Saale), Germany; 2 UR 406 Abeilles et Environnement, INRA, Avignon, France; 3 Department of Agroecology, Aarhus University, Flakkebjerg, Denmark; 4 Department of Internal Medicine IV, Martin-Luther-Universität Halle-Wittenberg, Halle (Saale), Germany; 5 German Institute for Integrative Biodiversity Research (iDiv), Leipzig, Germany; 6 University of Pretoria, Department of Zoology and Entomology, Pretoria, South Africa; University of Cologne, GERMANY

## Abstract

Apoptosis is not only pivotal for development, but also for pathogen defence in multicellular organisms. Although numerous intracellular pathogens are known to interfere with the host’s apoptotic machinery to overcome this defence, its importance for host-parasite coevolution has been neglected. We conducted three inoculation experiments to investigate in the apoptotic respond during infection with the intracellular gut pathogen *Nosema ceranae*, which is considered as potential global threat to the honeybee (*Apis mellifera*) and other bee pollinators, in sensitive and tolerant honeybees. To explore apoptotic processes in the gut epithelium, we visualised apoptotic cells using TUNEL assays and measured the relative expression levels of subset of candidate genes involved in the apoptotic machinery using qPCR. Our results suggest that *N*. *ceranae* reduces apoptosis in sensitive honeybees by enhancing *inhibitor of apoptosis protein-*(*iap*)*-2* gene transcription. Interestingly, this seems not be the case in *Nosema* tolerant honeybees. We propose that these tolerant honeybees are able to escape the manipulation of apoptosis by *N*. *ceranae*, which may have evolved a mechanism to regulate an anti-apoptotic gene as key adaptation for improved host invasion.

## Introduction

In insects, epithelial cells of the intestine are typically the first line of pathogen defence. They produce not only antimicrobial peptides (AMPs) and reactive oxygen species (ROS) but they can also respond with programmed cell death (including apoptosis) of infected cells. The infection may then be simply cleared by defecation. Hence, it is not surprising to see that intracellular pathogens have evolved mechanisms to overcome apoptosis for self-protection and increase of reproductive success within their host cell [1–3]. This is also the case for microsporidia [4–6], a group of highly specialised intracellular fungal parasites, causing diseases in a wide range of animal hosts, including humans and several animal species important for agriculture and aquaculture [7].

Although strategies of numerous pathogens have been studied in some detail [1–3], adaptations by the host to withstand the manipulation by these pathogen are neglected. The inhibition of apoptosis was recently shown to be pivotal for a successful infection of the microsporidian pathogen *Nosema ceranae* [5], which is considered to be a major threat to honeybees and wild bee pollinators [8–10]. Several honeybee transcriptome studies also indicated a link between *Nosema* spp. infections and apoptosis and epithelium renewal [11–13], supporting the idea that apoptosis might play a central role during *Nosema* infection. *Nosema* spores are transmitted via the faecal-oral route and germinate in the host midgut and enter epithelial cells, where they replicate and produce a new generation of spores after 4 days [9, 14].

Because Nosemosis can seriously impact colony health, Danish bee breeders successfully selected for *Nosema* resistant colonies, which appear to be the result of tolerance at the individual level [15, 16]. To examine the importance of the apoptotic defence system in the adaptation of these *Nosema* tolerant honeybees, we compared them with sensitive honeybees in three controlled inoculation experiments and screened for apoptotic processes in the honeybee midgut epithelium.

## Materials and Methods

### Experimental inoculation

One colony of *Nosema* tolerant honeybees from Aarhus (Denmark) was transported to Avignon (France), where one colony with *Nosema* sensitive honeybees was chosen for positive controls. We replicated three independent inoculation experiments in May 2013 following standard methods [17]. Briefly, newly emerged workers (< 24 h old) were collected from the brood frames. *Nosema* sensitive (SN) and tolerant (TN) honeybees were individually fed with 10^5^ freshly extracted and purified *N*. *ceranae* spores in 2 μl sucrose solution. Uninfected controls of the sensitive (SC) and tolerant (TC) honeybee strain were only fed with 2 μl sucrose solution. Individuals that have not consumed the inoculum were discarded from the experiment. Twenty worker bees per honeybee strain were housed in sterile stainless steel cages (10×10×5.5 cm) with a piece of clean wax foundation in an incubator at 34 ± 1°C, 60% relative humidity and provided with 50% (w/v) sucrose solution *ad libitum*. Bees were sacrificed either on one or six days post infection (p.i.). Their midguts (ventriculi without rectum) were dissected and stored accordingly to the analysis. We confirmed the treatment success by estimating the number of *Nosema* spp. spores for a random subset of 5 midgut samples for each treatment group and replicate using a Fuchs–Rosenthal haemocytometer under a phase-contrast microscope (×400).

### Immunohistochemistry

**Fig 1:**
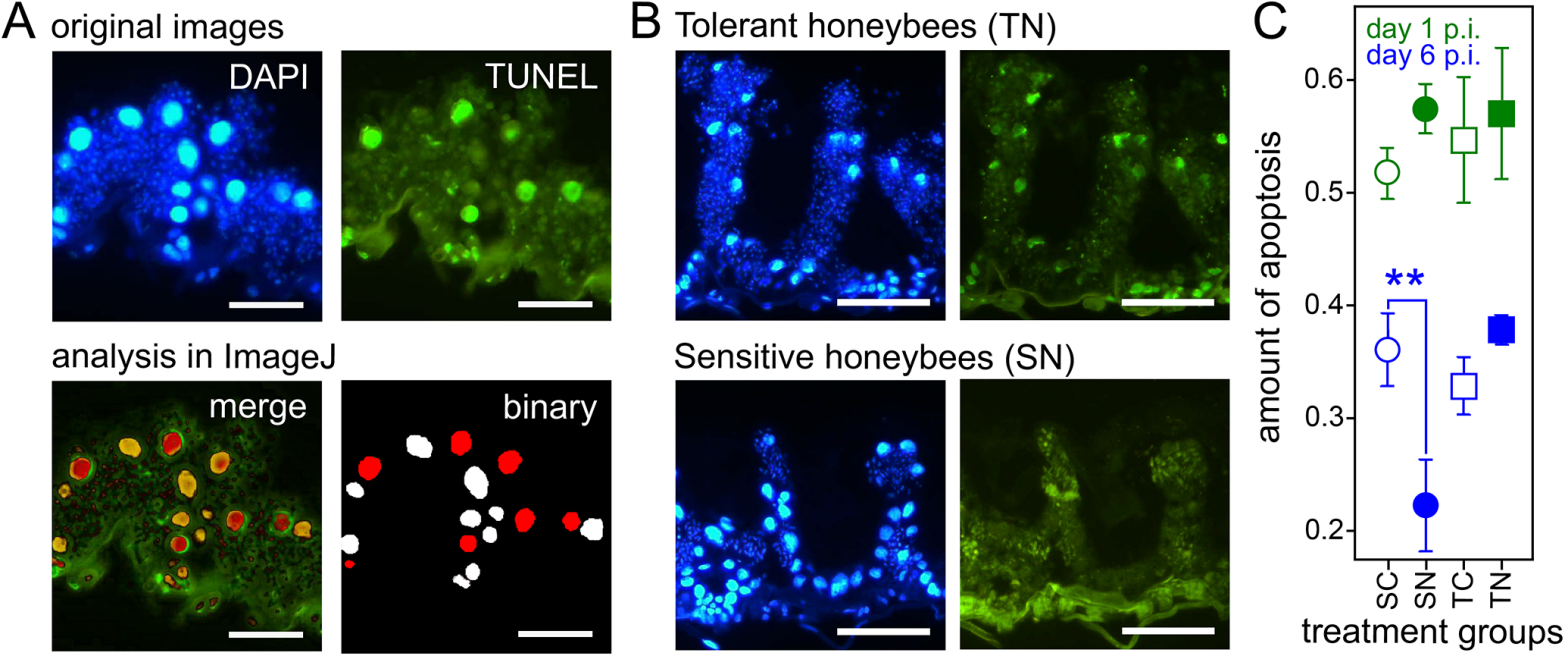
Quantification of apoptosis in the midgut epithelium of honeybees infected with *N*. *ceranae*. (A) The frequency of apoptotic cells was calculated as the numbers of TUNEL+ve relation to all (DAPI+ve) nuclei. For this, DAPI and TUNEL stained images (top) were merged (bottom left); nuclei were binarised and automatically counted using ImageJ (bottom right; red = TUNEL+ve, white = TUNEL–ve). Scale bars = 25 μm. (B) Comparison of apoptotic TUNEL+ve cells detected in the posterior end of the midgut in *Nosema* infected sensitive and tolerant honeybees on day 6 p.i. Scale bars = 50 μm. (C) Apoptosis ratio (mean ± s.e.) during *Nosema ceranae* infection in *Nosema* sensitive (SN, solid circles) and tolerant (TN, solid squares) honeybees, and their uninfected controls (SC, open circles and TC, open squares) at 1 day (green) and 6 days (blue) after inoculation. Sample sizes are given in S2 Table. Significance between treatment groups **, *P* < 0.01.

4% buffered formaldehyde (Süsse) was used for fixation of three midguts per replicate for 24 h at 8°C, followed by paraffin embedding according to standard histological methods. The ratio of apoptosis was determined by TUNEL (Terminal deoxynucleotide transferase mediated X-dUTP nick endlabelling) assays (In Situ Cell Death Detection Kit, Roche) on 7 μm thick longitudinal sections according to the manufacturer’s manual. This method allows the detection of apoptotic cells at the early stage, for which selective internucleosomal DNA degradation is characteristic, by directly labelling of single–and double–stranded DNA nicks with the enzyme TdT (Terminal deoxynucleotide transferase) and fluorescein–dUTP. Prior to the TUNEL reactions we blocked endogenous peroxidase activity (Dual Endogenous Enzyme Block, Dako), followed by permeabilisation step using nucleases–free 10 μg μl^–1^ proteinase K in 10 mM Tris/HCl pH 7.5 for 20 min at room temperature and rinsed the samples twice in PBS (phosphate–buffered saline). The TUNEL reaction was stopped after 1 h at 37°C in the dark by rinsing the samples three times in PBS and then counterstaining with 1 μg ml^−1^ DAPI (4',6-diamidino-2-phenylindole) (Sigma-Aldrich). We visualised apoptotic cells (TUNEL+ve) relative to the total number of cells (DAPI+ve) in the posterior part of midguts (primary site of the infection on day 6 p.i.) using fluorescence microscopy and acquired images with CCD camera connected to Axio Vision 4.6 (Zeiss). Automatic cell counting and analyses were performed with ImageJ [18] (Fig 1A) screening at the average 325 ± 16 s.e. cells per sample (see also S2 Table).

### Gene expression

Midguts of nine workers for each treatment group and for each replicated experiment were sampled in pools of three individuals, flash-frozen in liquid nitrogen and stored at -80°C until subsequent qPCR analyses. Briefly, total RNA was isolated using TRIZOL extraction procedure and 1 μg RNA each were reverse transcribed. Obtained cDNA were purified using QIAquick PCR Purification Kit (Qiagen). We performed a TBLASTN search [19] of *Apis mellifera* (taxid 7460) database using full-length amino acid sequences for key proteins involved in apoptosis known from *Drosophila melanogaster* [20]. Only homologous proteins with at least 20% identity were considered for this study (S1 Table). Thus some proteins known to be relevant for apoptosis in *D*. *melanogaster* were not included due to insufficient homology. Gene specific primers spanning one intron of subset of nine potential candidate genes, including the genes *basket* (*bsk*), *tumor protein p53-like* (*p53*), *inhibitor of apoptosis protein 2* (*iap–2; homologous gene to Diap–1*), *caspase-2-like* (*casp–2; possible homologous gene to Dronc*) and *caspase-10-like* (*casp–10; homologous gene to Dredd*), were designed using Primer-BLAST on the *A*. *mellifera* reference genome (release v.4.5, GenBank, S1 Table). *Ribosomal protein S5a* (*RpS5a*) and *actin related protein 1* (*arp1*, also known as *actin*) were initially chosen in order to standardize expression levels between pools and treatment groups [21]. For qPCR, we used 20 ng cDNA mixed with 5 μl SensiMixPlus (Bioline), 0.25 μM of each primer and DEPC-water in 10 μl final volume. Initial denaturation at 95°C for 10 min was followed by 40 amplification cycles (95°C for 15 s, 59°C for 30 s, 72°C for 30 s), ending with melting curve analysis from 50°C to 98°C in 1°C increments. At least two technical replicates were run per sample using Chromo4 ™ (Bio–Rad) and repeated if necessary to obtain a delta C_t_ (threshold cycle) value below 0.5 between two replicates (LinRegPCR [22]). Correct amplicon sizes absence of non-specific products were verified using the high-resolution automatic capillary electrophoresis system QIAxcel^®^ (Qiagen). We tested the suitability of *RpS5a* and *arp1* as previously described [23], and found the housekeeping gene *RpS5a* (s.d. = 0.86) to be more suitable for normalisation of gene expression levels among samples, and thus excluded the *arp1* (s.d. = 1.55) from further analyses.

### Statistics

All statistical analyses and data plotting (mean ± s.e.) were performed in R (v.3.0.2) [24]. Spore load between infected groups was tested using Welch’s two-tailed *t*-test. We used Generalized Linear Models (GLM) based on quasilikelihood estimation with a binomial error distribution to test the effects of honeybee strain and treatment and their interactions on the apoptosis ratio for each day p.i. independently. Post-hoc analyses were performed using the Tukey's HSD. The effects of day p.i., honeybee strain and treatment and their interactions on relative gene expression were tested using linear models (LM) for each gene separately, accounting for multiple testing with Bonferroni adjustments. We used the likelihood ratio test to test single parameters and their interactions, comparing the goodness–of–fit between the models [25]. If a model was found to be unstable with all interactions included, we removed non–significant interactions step-wise. Model validity was tested by comparing full models to their null models without any fixed factors included using likelihood ratio test. Tukey's HSD post-hoc contrast analyses were performed using the glht function with Bonferroni adjustment (multcomp package, v.1.3–2.).

## Results and Discussion

There were generally higher apoptosis rates in all four treatment groups tested on day 1 p.i. than on day 6 p.i. (GLM: day, estimate ± s.e. = -0.904 ± 0.239, *P* < 0.001, Fig 1B), suggesting that midgut epithelial cells in those young bees were still undergoing morphogenetic developments at age of 1–2 day [26]. More interestingly, however, was the interaction between treatment and honeybee strain (GLM: -0.899 ± 0.293, *P* < 0.005; Fig 1B) whereby *N*. *ceranae* infection reduced the rate of apoptosis in sensitive honeybees (Tukey's HSD: -0.679 ± 0.210, *P* < 0.007), confirming the suppression of apoptosis by microsporidia [5], but interestingly this was not the case in the infected tolerant honeybees (Tukey's HSD: 0.220 ± 0.205, *P* = 1). Hence, these results suggest an adaptive mechanism in the tolerant honeybees which enables them to remove infected cells from the epithelia into the gut lumen and presumably to eventually defecate.

As honeybees usually defecate during short defecation flights and avoid defecation in their nest [27], infected apoptotic cells have presumably been accumulated in the midgut and rectum in *Nosema* tolerant honeybees at this early stage of the infection in our cage experiments. This in fact would be a plausible explanation why we have not counted less numbers of *Nosema* spores between sensitive (SN: 6.0 ± 1.2*×*10^6^ spores; *n* = 14) and tolerant honeybees (TN: 8.0 ± 1.4*×*10^6^ spores; *n* = 15) on day 6 p.i. (*t-test*: *t* = 1.115, *d*.*f*. = 26.77, *P* < 0.275).

**Fig 2:**
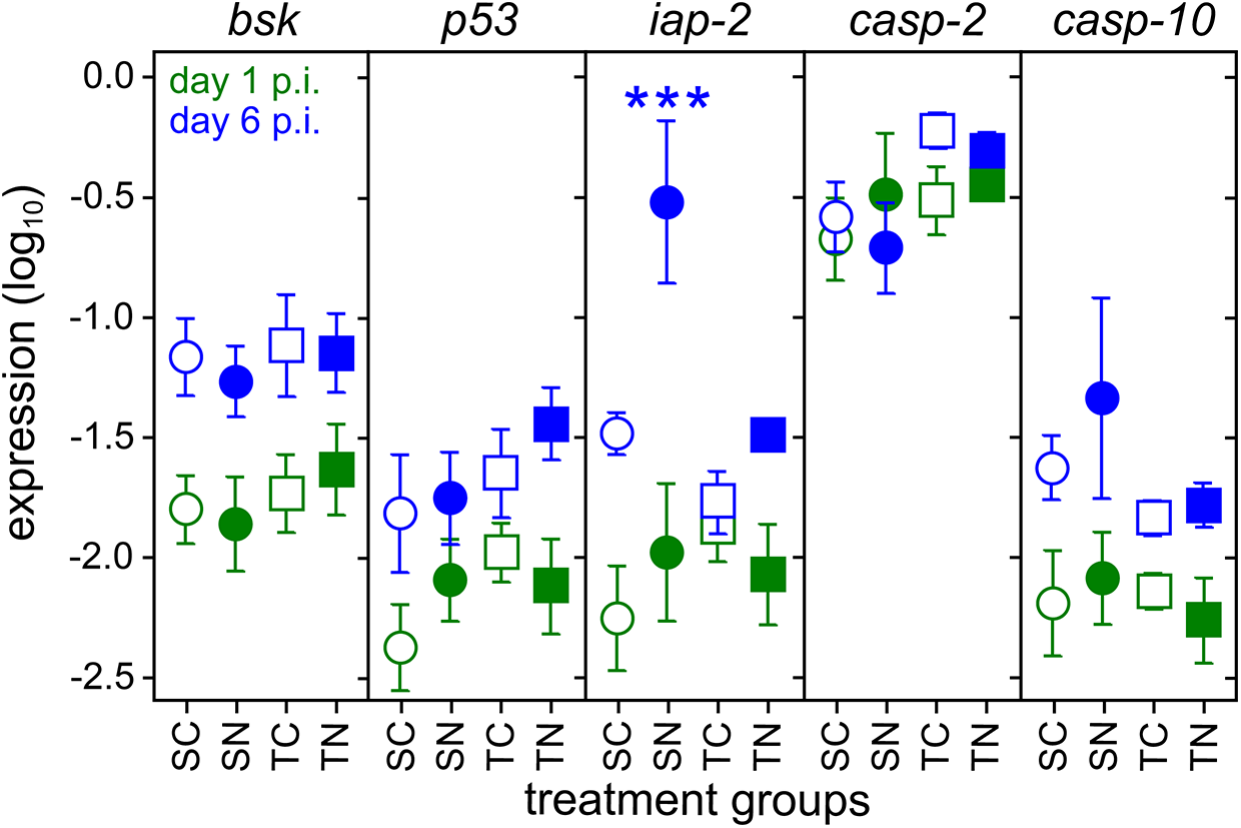
Relative expression (mean ± s.e.) of candidate genes important for apoptosis in *Nosema* infected honeybees. *Nosema* sensitive (SN, solid circles) and tolerant (TN, solid squares) honeybees infected with 10^5^ *N*. *ceranae* spores, and their controls uninfected (SC, open circles and TC, open squares), were sampled at 1 day (green) and 6 days (blue) after inoculation. The genes JNK/*bsk* (Jun N–terminal kinase/ *basket*), *p53* (*tumor protein p53-like*), *iap–2* (*inhibitor of apoptosis protein 2;* predicted homologous gene to *Diap–1* in *D*. *melanogaster*), *casp–2* (*caspase–2–like; homologous gene to Dcp–1*), *casp–10* (*caspase–10–like; homologous gene to Dredd*) were predicted from *Drosophila melanogaster*. Sample sizes are ranging between six and ten pools of three individual honeybee midguts (see also S3 Table). Significance between treatment groups ***, *P* < 0.001.

To explore the molecular mechanisms underlying the inhibition of apoptosis in the sensitive honeybee, we measured relative gene expression levels of candidate genes in the apoptotic cascade predicted from *Drosophila* [20] (S1 Table). We found age–related alterations in gene expression levels for Jun N–terminal kinase (JNK)/*basket* (*bsk*, linear model (LM): day p.i., 0.091 ± 0.026, *P* < 0.007; Fig 2) and *tumor suppressor protein p53* (*p53*, LM: day p.i., 0.022 ± 0.005, *P* < 0.001; Fig 2), which were slightly higher expressed on day 6 p.i. than on day 1 p.i. over all treatment groups. Although both JNK/*bsk* and *p53* are important proapoptotic factors [28, 29], the core of the apoptotic–machine consists of caspases that destroy essential cell proteins that initiate apoptosis [20, 30, 31]. Surprisingly, *caspase–10–like* (homologous gene to *Dredd* in *D*. *melanogaster*) was slightly higher expressed in the sensitive honeybees regardless the treatment than in the tolerant honeybees on day 6 p.i. (*casp–10*, LM: honeybee strain, 0.286 ± 0.093, *P* < 0.025; Fig 2). The *caspase–2–like* gene (possible homologous gene to *Dronc* in *D*. *melanogaster*), however, was not differentially expressed between treatment groups (*casp–2*, LM: 0.074 ± 0.074, *P* < 0.290; Fig 2). In *D*. *melanogaster* the apical cell-death caspase DRONC, mediated by the adapter ARK (homolog to the apoptotic protease-activating factor 1, *Apaf 1*), plays a central role due to its chronic activation in many cells [20]. This may also be the case for *casp–2* in honeybees as we measured relatively high expression levels in all treatment groups. Nevertheless, cells survive due to the DIAP1 expression, which suppresses DRONC and other activated caspases [20, 32]. Interestingly, we found an interaction between honeybee strain and treatment on day 6 p.i. (LM: honeybee strain *×* treatment: 0.751 ± 0.260, *P* < 0.038; Fig 2), whereby *iap–2* (predicted homologous gene of *Diap–1* in *D*. *melanogaster*) expression appeared to be tenfold increased on average in sensitive honeybees when infected with *N*. *ceranae* (Tukey's HSD: 0.761 ± 0.198, *P* < 0.001) but we found no alterations in *iap–2* expression level in tolerant honeybees (Tukey's HSD: 0.010 ± 0.169, *P* < 0.980).

Although IAPs are also known to play a regulatory role in pathogen-sensing pathways and induction of the innate immune system [20, 32–34], we speculate that the up-regulation of *iap–2* in *Nosema* infected sensitive honeybees might be rather involved in cell survival, because previous studies reported that *N*. *ceranae* causes immunosuppression in sensitive honeybees [16, 35, 36]. Furthermore, this up-regulation of *iap–2* only in *Nosema* infected sensitive honeybees would also plausibly explain the reduced apoptosis activity in sensitive honeybees in our TUNEL assays and supports previous findings [5]. Unfortunately, we did not measure the expression levels of potential apoptosis inducing proteins such as Reaper, Hid and Grim (known as RHG proteins), which can negatively regulate DIAP1 activity in *D*. *melanogaster* [20, 34]. Nevertheless, *in vitro* studies have demonstrated in some more detail that infections with protozoans such as *Toxoplasma gondii* [37], *Cryptosporidium parvum* [38] as well as bacteria *Shigella flexneri* [39] and *Neisseria gonorrhoeae* [40] elicit up-regulation of *iap* genes and result in the inhibition of host cell apoptosis in mammalian host cell cultures. The activation of the key transcription factor NF-кB was shown to correlate with *iap* transcription [37, 40] and may also be the case in our *in situ* honeybee-*Nosema* system and might be triggered by elevated *Nosema* HSP70 levels [41, 42]. The capability to retain high apoptotic activity in spite of a *Nosema* infection might explain why the tolerant honeybees can overcome the infection and eliminate the disease from the colony [15, 16]. Workers presumably simply clear the infection by removing those apoptotic infected cells on defecation flights. In contrast sensitive honeybees might be likely to retain the infection in the gut epithelium for much longer time. The dynamics of intestine epithelium development [26] may also provide an alternative explanation for the age-dependent *Nosema* susceptibility in *Bombus terrestris* and honeybees previously exclusively attributed to age polyethism [8, 43].

Our results provide a snap shot of host-parasite co-evolution, where artificial selection of the honeybee host has presumably accelerated a counter adaptation towards *Nosema*. Irrespective of the actual molecular mechanisms, this study does not only highlight the central role of apoptosis for host immunity in general but also shows the importance of its manipulation for intracellular pathogens. Understanding the molecular dialogue between infecting pathogen and host cell might not only be interesting for evolutionary biology and parasitology, but may also provide novel perspectives for effective immunological strategies in the treatment of animal and human diseases by interfering into the regulatory machinery of apoptosis.

## Supporting Information

S1 Table Primer sequences used in qPCR. (DOCX)

S2 Table Data of the estimation of the apoptosis rate in the posterior end of honeybee midguts for days one and six post infection (d.p.i). (DOCX)

S3 Table Relative gene expression data of predicated candidate genes (*bsk*, *p53*, *iap–2*, *casp–2* and *casp–10*). (DOCX)
