# Supplementary material for: Nosema Tolerant Honeybees (Apis mellifera) Escape Parasitic Manipulation of Apoptosis
Source: PLoS One. 2015 Oct 7;10(10):e0140174. doi: 10.1371/journal.pone.0140174 (PMC4596554; doi:10.1371/journal.pone.0140174)
Supplement: S1 Table — (DOCX) [file pone.0140174.s001.docx]

**S1 Table.** Primer sequences used in qPCR.

| **Gene** | **Predicted in *Apis mellifera*** | **Homolog in *D. melanogaster*** | **GenBank assacion no.** | **Primer sequences** | | **References** |
| --- | --- | --- | --- | --- | --- | --- |
| *ark* | *apoptotic protease-activating factor (Apaf) 1-like* | *Apaf-1-related-killer*, isoform B | XR_120278.1 | forward, CACCCACTCTTCCTCCAACAT;  reverse, ATATTTCTTCATCAACTGGACGTTT | this study | |
| *bsk* | *JNK MAP kinase basket* | *basket*, isoform B | XM_392806.4 | forward, ACAAGATAATCGAGCAACTGGGA;  reverse, CCTGTTCTCCACGTAATTCCTGA | this study | |
| *bcl-2* | *bcl-2-related ovarian killer protein* | *Bcl-2 related protein* (*buffy*) | XM_395083.4 | forward, GCATTGCCGATGCCTGAAAA;  reverse, TCTGCGATAAGGTTGGCCTG | this study | |
| *casp-10* | *caspase-10-like* (LOC724930) | *death related ced-3/Nedd2-like protein (Dredd)** | XM_001120830.2 | forward, GCGTCATAAAGAAAAAGGATCATGG; reverse, CCCCTACTGCATCAATTGTTTT | this study | |
| *casp-1* | *caspase-1-like* (LOC412235) | *Dredd*, Nedd2-like caspase (Dronc)** | XM_395697.4 | forward, CCGCGAGTGGTATTTCTCCA;  reverse, ACTGATGGTCAACCAGCTTCT | this study | |
| *casp-2* | *caspase-2-like* | *Dronc** | XM_003249125.1 | forward, TCACGTTGGAAGACAAATCTCAC;  reverse, AATGCAAAAGGTCCCCGTGT | this study | |
| *casp* | *caspase-like* (LOC411381) | *Drice*; Dronc** | XM_394855.3 | forward, ACAGTATGACGGATGCCGAG;  reverse, GGCGAATTTCTTCTGTGCATGT | this study | |
| *iap-2* | *inhibitor of apoptosis protein 2* | *apoptosis 1 inhibitor* (*Diap-1*) | XM_396819.4 | forward, TTCAACGTGATGATGAAGTACAATG;  reverse, TGGATCATCACCAAGCTCCC | this study | |
| *p53* | *tumor protein p73-like* | *tumor protein* *p53* | XM_003249744.1 | forward, TGGAATATGACCACTACGAATGGA;  reverse, AGGGTACAAATCCAATATCCTTCAC | this study | |
|  |  |  |  |  |  | |
| *arp 1* | *actin related protein 1* |  | NM_001185146.1 | forward, TTGTATGCCAACACTGTCCTTT;  reverse, TGGCGCGATGATCTTAATTT | [[1](#_ENREF_1)] | |
| *RPS5* | *ribosomal protein S5a* |  | XM_624081.3 | forward, AATTATTTGGTCGCTGGAATTG;  reverse, TAACGTCCAGCAGAATGTGGTA | [[1](#_ENREF_1)] | |

* Due to high sequence similarities, a distinction between caspases, as known for *D. melanogaster*, was difficult in *Apis mellifera* based on gene homologies*.*

**Additional References**

1. Evans JD (2006) Beepath: An ordered quantitative-PCR array for exploring honey bee immunity and disease. J Invertebr Pathol 93: 135-139.
