# Supplementary material for: Nosema Tolerant Honeybees (Apis mellifera) Escape Parasitic Manipulation of Apoptosis
Source: PLoS One. 2015 Oct 7;10(10):e0140174. doi: 10.1371/journal.pone.0140174 (PMC4596554; doi:10.1371/journal.pone.0140174)
Supplement: S2 Table — (DOCX) [file pone.0140174.s002.docx]

**S2 Table.** Data of the estimation of the apoptosis rate in the posterior end of honeybee midguts for days one and six post infection (d.p.i). The apoptosis ratio was calculated as TUNEL+ve / DAPI+ve nuclei using ImageJ. For day 1 p.i.: *n* = 3_SC_, 5_SN_, 5_TC_, 6_TN_ and for day 6 p.i.: *n* = 5_SC_, 7_SN_, 9_TC_, 7_TN._

| **#ID** | **treatment groups** | **treatment** | **group** | **d.p.i.** | **replicate** | number of nuclei | **apoptosis**  **ratio** |
| --- | --- | --- | --- | --- | --- | --- | --- |
| 1 | SN | infected | sensitive | six | 1 | 354 | 0.362 |
| 2 | SN | infected | sensitive | six | 1 | 457 | 0.225 |
| 3 | SN | infected | sensitive | six | 1 | 273 | 0.348 |
| 4 | SC | control | sensitive | six | 1 | 328 | 0.451 |
| 5 | SC | control | sensitive | six | 1 | 342 | 0.471 |
| 6 | SC | control | sensitive | six | 1 | 384 | 0.477 |
| 7 | TN | infected | tolerant | six | 1 | 515 | 0.373 |
| 8 | TN | infected | tolerant | six | 1 | 624 | 0.404 |
| 9 | TN | infected | tolerant | six | 1 | 516 | 0.341 |
| 10 | TN | infected | tolerant | six | 1 | 538 | 0.394 |
| 11 | TC | control | tolerant | six | 1 | 345 | 0.304 |
| 12 | TC | control | tolerant | six | 1 | 293 | 0.294 |
| 13 | TC | control | tolerant | six | 1 | 283 | 0.339 |
| 14 | SN | infected | sensitive | six | 3 | 483 | 0.178 |
| 15 | SN | infected | sensitive | six | 3 | 362 | 0.224 |
| 16 | SC | control | sensitive | six | 3 | 376 | 0.314 |
| 17 | SC | control | sensitive | six | 3 | 362 | 0.304 |
| 18 | SC | control | sensitive | six | 3 | 407 | 0.405 |
| 20 | TN | infected | tolerant | six | 3 | 341 | 0.375 |
| 21 | TC | control | tolerant | six | 3 | 467 | 0.272 |
| 22 | TC | control | tolerant | six | 3 | 342 | 0.398 |
| 24 | SN | infected | sensitive | six | 2 | 429 | 0.054 |
| 25 | SN | infected | sensitive | six | 2 | 359 | 0.167 |
| 26 | SC | control | sensitive | six | 2 | 417 | 0.223 |
| 27 | SC | control | sensitive | six | 2 | 267 | 0.228 |
| 28 | SC | control | sensitive | six | 2 | 296 | 0.375 |
| 29 | TN | infected | tolerant | six | 2 | 297 | 0.387 |
| 30 | TN | infected | tolerant | six | 2 | 436 | 0.374 |
| 31 | TC | control | tolerant | six | 2 | 371 | 0.251 |
| 32 | TC | control | tolerant | six | 2 | 431 | 0.439 |
| 33 | SN | infected | sensitive | one | 1 | 70 | 0.629 |
| 34 | SN | infected | sensitive | one | 1 | 343 | 0.615 |
| 35 | SN | infected | sensitive | one | 3 | 288 | 0.580 |
| 43 | TC | control | tolerant | one | 3 | 297 | 0.401 |
| 44 | SN | infected | sensitive | one | 2 | 204 | 0.515 |
| 45 | SN | infected | sensitive | one | 2 | 306 | 0.533 |
| 46 | SC | control | sensitive | one | 2 | 364 | 0.492 |
| 47 | SC | control | sensitive | one | 2 | 187 | 0.561 |
| 48 | SC | control | sensitive | one | 3 | 194 | 0.500 |
| 49 | TN | infected | tolerant | one | 2 | 175 | 0.617 |
| 50 | TN | infected | tolerant | one | 2 | 372 | 0.540 |
| 51 | TN | infected | tolerant | one | 2 | 91 | 0.835 |
| 52 | TC | control | tolerant | one | 2 | 310 | 0.529 |
| 53 | TC | control | tolerant | one | 2 | 165 | 0.727 |
| 57 | TN | infected | tolerant | one | 3 | 174 | 0.540 |
| 58 | TN | infected | tolerant | one | 1 | 253 | 0.443 |
| 59 | TN | infected | tolerant | one | 1 | 235 | 0.447 |
| 60 | TC | control | tolerant | one | 1 | 186 | 0.608 |
| 62 | TC | control | tolerant | one | 1 | 218 | 0.468 |
