# Supplementary material for: Nosema Tolerant Honeybees (Apis mellifera) Escape Parasitic Manipulation of Apoptosis
Source: PLoS One. 2015 Oct 7;10(10):e0140174. doi: 10.1371/journal.pone.0140174 (PMC4596554; doi:10.1371/journal.pone.0140174)
Supplement: S3 Table — (DOCX) [file pone.0140174.s003.docx]

**S3 Table.** Relative gene expression data of predicated candidate genes (*bsk*, *p53*, *iap-2*, *casp-2* and *casp-10*) involved in apoptosis in *Nosema* *ceranae* infected *Nosema* sensitive (SN) and tolerant (TN) honeybees, and their uninfected controls (SC, TC) after one and six days post infection (d.p.i.). Sample ID represents pools of midguts from three individual honeybees.

| **#ID** | **treatment groups** | **treatment** | **group** | **d.p.i.** | **replicate** | ***bsk*** | ***p53*** | ***iap-2*** | ***casp-2*** | ***casp-10*** |
| --- | --- | --- | --- | --- | --- | --- | --- | --- | --- | --- |
| 1 | TN | infected | tolerant | one | 1 | 0,009 | NA | 0,010 | 0,505 | 0,003 |
| 2 | TC | control | tolerant | one | 1 | 0,003 | 0,016 | 0,012 | 0,736 | 0,009 |
| 4 | SC | control | sensitive | one | 1 | 0,009 | 0,001 | 0,001 | 0,126 | NA |
| 5 | TN | infected | tolerant | one | 2 | 0,017 | 0,001 | 0,002 | 0,168 | 0,002 |
| 6 | TC | control | tolerant | one | 2 | 0,008 | 0,016 | 0,024 | 0,585 | 0,010 |
| 7 | TC | control | tolerant | one | 3 | 0,011 | 0,002 | 0,002 | 0,056 | 0,003 |
| 8 | SN | infected | sensitive | one | 2 | 0,004 | 0,008 | 0,011 | 1,193 | 0,010 |
| 9 | SC | control | sensitive | one | 2 | 0,009 | 0,002 | 0,006 | 0,415 | 0,003 |
| 10 | TN | infected | tolerant | one | 3 | 0,006 | 0,016 | 0,015 | 0,432 | 0,010 |
| 11 | SN | infected | sensitive | one | 3 | 0,007 | 0,021 | 0,088 | 3,424 | 0,013 |
| 12 | SC | control | sensitive | one | 3 | 0,009 | NA | 0,002 | 0,086 | 0,004 |
| 23 | TN | infected | tolerant | six | 1 | 0,072 | 0,009 | 0,033 | 0,454 | 0,028 |
| 24 | TC | control | tolerant | six | 1 | 0,708 | 0,027 | 0,034 | NA | 0,017 |
| 25 | SN | infected | sensitive | six | 1 | 0,132 | 0,020 | 1,471 | 0,680 | 0,035 |
| 26 | SC | control | sensitive | six | 1 | 0,018 | 0,010 | 0,015 | 0,144 | NA |
| 27 | TN | infected | tolerant | six | 1 | 0,276 | NA | NA | 0,829 | NA |
| 28 | TC | control | tolerant | six | 2 | NA | 0,025 | 0,028 | 1,049 | 0,019 |
| 29 | TN | infected | tolerant | six | 3 | 0,074 | 0,032 | 0,024 | 0,717 | 0,006 |
| 30 | TC | control | tolerant | six | 3 | 0,072 | 0,020 | 0,017 | 0,612 | 0,005 |
| 31 | SN | infected | sensitive | six | 3 | NA | NA | NA | NA | NA |
| 32 | SC | control | sensitive | six | 3 | NA | 0,024 | 0,034 | 0,219 | NA |
| 33 | TN | infected | tolerant | six | 2 | 0,065 | 0,031 | 0,028 | 0,617 | 0,016 |
| 34 | TC | control | tolerant | six | 2 | 0,015 | NA | 0,014 | NA | NA |
| 35 | SN | infected | sensitive | six | 2 | 0,033 | 0,002 | 0,307 | 0,128 | 0,001 |
| 36 | SC | control | sensitive | six | 2 | 0,048 | 0,001 | NA | 0,446 | 0,012 |
| 37 | TN | infected | tolerant | one | 1 | 0,034 | 0,017 | 0,017 | 0,243 | 0,015 |
| 38 | TC | control | tolerant | one | 1 | 0,015 | 0,014 | 0,014 | 0,489 | 0,010 |
| 39 | SN | infected | sensitive | one | 1 | 0,038 | 0,010 | 0,024 | 0,445 | 0,014 |
| 40 | SC | control | sensitive | one | 1 | 0,020 | 0,014 | 0,029 | 0,443 | 0,017 |
| 41 | TN | infected | tolerant | six | 1 | 0,022 | NA | 0,031 | 0,267 | 0,018 |
| 42 | TC | control | tolerant | six | 1 | 0,032 | 0,019 | 0,027 | 0,374 | 0,009 |
| 43 | SN | infected | sensitive | six | 1 | 0,034 | 0,016 | 0,030 | 0,411 | 0,471 |
| 44 | SC | control | sensitive | six | 1 | 0,171 | 0,019 | 0,088 | NA | 0,056 |
| 45 | TN | infected | tolerant | six | 3 | 0,078 | 0,130 | 0,037 | 0,353 | 0,014 |
| 46 | TC | control | tolerant | six | 3 | NA | NA | 0,032 | NA | NA |
| 47 | SN | infected | sensitive | six | 3 | 0,172 | NA | NA | 0,420 | 0,017 |
| 48 | SC | control | sensitive | six | 3 | 0,042 | NA | 0,039 | 0,083 | 0,011 |
| 49 | TC | control | tolerant | six | 1 | NA | 0,002 | 0,002 | 0,756 | 0,020 |
| 51 | TC | control | tolerant | six | 3 | 0,100 | NA | 0,021 | NA | 0,014 |
| 52 | TN | infected | tolerant | six | 2 | 0,104 | 0,045 | 0,033 | 0,305 | 0,033 |
| 53 | TN | infected | tolerant | six | 2 | NA | NA | NA | NA | NA |
| 54 | TN | infected | tolerant | six | 1 | 0,307 | 0,041 | 0,033 | 0,936 | NA |
| 55 | SC | control | sensitive | six | 3 | 0,152 | 0,048 | 0,034 | 0,805 | 0,064 |
| 56 | SC | control | sensitive | six | 2 | 0,234 | 0,057 | 0,021 | NA | 0,017 |
| 57 | SC | control | sensitive | six | 2 | NA | NA | NA | NA | NA |
| 58 | TC | control | tolerant | six | 2 | 0,054 | 0,075 | 0,004 | 0,685 | 0,016 |
| 59 | SN | infected | sensitive | six | 2 | NA | 0,029 | 2,035 | NA | NA |
| 60 | SN | infected | sensitive | six | 3 | NA | NA | NA | NA | NA |
| 61 | SN | infected | sensitive | six | 2 | 0,023 | 0,032 | 0,030 | 0,062 | 0,049 |
| 62 | TN | infected | tolerant | six | 3 | 0,009 | NA | 0,043 | 0,629 | 0,016 |
| 63 | TC | control | tolerant | six | 3 | 0,448 | NA | 0,045 | 0,291 | 0,014 |
| 64 | TC | control | tolerant | six | 2 | 0,020 | 0,060 | NA | 0,773 | 0,032 |
| 65 | SC | control | sensitive | six | 1 | 0,032 | 0,022 | 0,030 | 0,349 | 0,022 |
| 66 | SN | infected | sensitive | six | 1 | 0,042 | 0,047 | 0,917 | 0,059 | 0,696 |
| 84 | TN | infected | tolerant | one | 2 | 0,052 | 0,009 | 0,017 | 0,357 | 0,013 |
| 70 | TN | infected | tolerant | one | 3 | 0,082 | 0,014 | NA | 0,333 | 0,014 |
| 71 | SC | control | sensitive | one | 3 | 0,059 | 0,007 | 0,019 | 0,294 | 0,020 |
| 72 | SN | infected | sensitive | one | 3 | 0,041 | 0,012 | 0,012 | 0,268 | 0,013 |
| 73 | TC | control | tolerant | one | 3 | 0,048 | 0,019 | 0,013 | 0,305 | NA |
| 74 | SN | infected | sensitive | one | 2 | 0,023 | 0,015 | 0,008 | 0,191 | NA |
| 75 | SC | control | sensitive | one | 2 | 0,016 | 0,007 | 0,010 | 0,262 | 0,010 |
| 76 | TN | infected | tolerant | one | 2 | 0,074 | 0,008 | 0,014 | 0,348 | 0,009 |
| 77 | SC | control | sensitive | one | 2 | 0,043 | 0,009 | 0,017 | 0,761 | 0,020 |
| 78 | TN | infected | tolerant | one | 3 | 0,085 | 0,023 | 0,018 | 0,553 | 0,007 |
| 79 | TN | infected | tolerant | one | 2 | 0,001 | 0,001 | 0,000 | NA | 0,000 |
| 80 | SN | infected | sensitive | one | 1 | 0,021 | 0,010 | 0,020 | 0,421 | 0,015 |
| 81 | TC | control | tolerant | one | 2 | 0,047 | 0,010 | 0,014 | 0,212 | 0,006 |
| 82 | SN | infected | sensitive | one | 2 | 0,047 | 0,010 | NA | 0,285 | 0,013 |
| 83 | TC | control | tolerant | one | 3 | 0,060 | 0,016 | 0,044 | 0,318 | 0,011 |
| 85 | TC | control | tolerant | one | 2 | 0,026 | 0,007 | 0,016 | NA | 0,006 |
| 86 | SN | infected | sensitive | one | 3 | 0,001 | 0,001 | 0,000 | 0,010 | 0,001 |
| 87 | SC | control | sensitive | one | 3 | 0,037 | 0,011 | NA | 0,443 | 0,012 |
| 88 | TN | infected | tolerant | one | 1 | 0,044 | 0,024 | 0,029 | 0,455 | 0,010 |
| 89 | SC | control | sensitive | one | 3 | 0,003 | 0,001 | 0,001 | 0,016 | 0,000 |
